# Chemical and Structural Engineering of Guide RNAs for Precision Genome Editing: From Design Principles to Clinical Applications

**DOI:** 10.1111/cbdd.70377

**Published:** 2026-08-23

**Authors:** Masaki Kawamata, Satoshi Niwa, Atsushi Suzuki

**Affiliations:** ^1^ Convergence Research Promotion Office, Research Institute for Cell Design Medical Science (RICeD) Yamaguchi University Ube Yamaguchi Japan; ^2^ Division of Organogenesis and Regeneration, Medical Institute of Bioregulation Kyushu University Fukuoka Japan; ^3^ Department of Oral and Maxillofacial Surgery Yamaguchi University Graduate School of Medicine Ube Yamaguchi Japan

**Keywords:** CRISPR‐Cas9, fine‐tuning activity, genome editing, gRNA modification, off‐target

## Abstract

CRISPR–Cas9 has revolutionised genome editing by enabling efficient and programmable modification of defined DNA sequences, with guide RNAs (gRNAs) serving as indispensable elements that direct Cas9 to specific genomic loci. Initially regarded as auxiliary components, gRNAs are now recognized as critical determinants of editing efficiency and specificity and have attracted growing attention as independent targets for engineering. Chemical modification, sequence optimisation, and structural alteration of gRNAs have been shown to enhance on‐target activity, suppress off‐target effects and cytotoxicity, and even achieve allele‐selective precision editing in a programmable manner. Moreover, advances in artificial intelligence and machine learning have markedly improved the predictive accuracy of gRNA design through large‐scale data analysis. Despite rapid progress, a consolidated review that integrates chemical, structural, and computational advances in gRNA engineering and highlights their translational potential for therapeutic genome editing has been lacking. This review uniquely addresses that gap by presenting an integrated framework that connects molecular design principles with clinical applicability.

## Graphical abstract

Chemical and structural engineering strategies enhance gRNA stability, activity, specificity, and translational potential for precision genome editing. This review summarizes recent advances, design principles, and emerging clinical applications of engineered gRNAs.

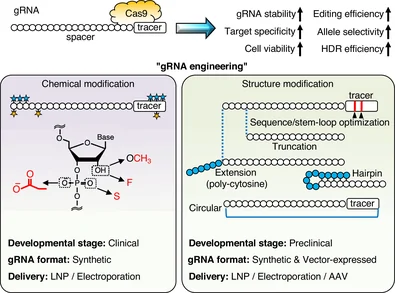

## 1 Introduction

CRISPR–Cas9, adapted from the bacterial immune system, is a powerful genome‐editing tool that enables the rewriting of defined DNA sequences through the induction of targeted double‐strand breaks (DSBs) at specific genomic sites (Cho et al. 2013; Cong et al. 2013; Jinek et al. 2012; Mali et al. 2013). The system consists of two key components: the Cas9 nuclease, which cleaves DNA, and the guide RNA (gRNA). The gRNA is typically engineered as a single chimeric RNA composed of a ~20‐nucleotide spacer sequence derived from CRISPR RNA (crRNA), which confers sequence specificity, and a scaffold derived from trans‐activating CRISPR RNA (tracrRNA), which interacts with Cas9. The gRNA sequence is complementary to the selected DNA target, thereby directing Cas9 to the corresponding genomic locus. Once recruited, Cas9 introduces a DSB at the target site, which is subsequently repaired by the cell's endogenous DNA repair machinery. In mammalian cells, DSBs are primarily repaired through the non‐homologous end‐joining (NHEJ) pathway (Lieber 2010; Mao et al. 2008), leading to efficient generation of knockout alleles through the induction of frameshift mutations (Shalem et al. 2014). By contrast, the alternative pathway of homology‐directed repair (HDR) can be exploited for precise genetic modifications, such as codon replacements using exogenous DNA oligonucleotides or vectors that serve as repair templates (San Filippo et al. 2008).

Unlike earlier protein‐based genome‐editing tools such as zinc‐finger nucleases (ZFNs) and transcription activator‐like effector nucleases (TALENs), CRISPR–Cas9 enables flexible and cost‐effective target switching simply by altering the gRNA sequence while maintaining high editing efficiency (Guo et al. 2023). However, Cas9‐mediated cleavage also carries the risk of unintended mutations at off‐target sites with sequence similarity to the intended target. Such off‐target cleavage increases the overall burden of DNA double‐strand breaks (DSBs) within cells, thereby elevating the risk of genomic instability. Excessive DSB formation can lead to undesirable outcomes, including large deletions, chromosomal rearrangements, and activation of DNA damage response pathways. In particular, the accumulation of multiple DSBs has been associated with p53‐mediated cell cycle arrest or apoptosis, potentially reducing editing efficiency and impairing the recovery of successfully edited cells (Haapaniemi et al. 2018; Ihry et al. 2018; Kosicki et al. 2018). Therefore, minimizing off‐target activity through rational gRNA engineering is critical not only for improving editing specificity but also for reducing DSB‐associated cellular toxicity and genomic alterations.

These challenges represent major obstacles to the safe clinical translation of CRISPR technology. To address these limitations, several high‐fidelity Cas9 variants have been engineered through amino acid substitutions, including eSpCas9(1.1) (Slaymaker et al. 2016), SpCas9‐HF1 (Kleinstiver et al. 2016), HypaCas9 (Chen et al. 2017), evoCas9 (Casini et al. 2018), Sniper‐Cas9 (Lee et al. 2018), HiFi Cas9 (Vakulskas et al. 2018), Opti‐SpCas9 (Choi et al. 2019), LZ3 Cas9 (Schmid‐Burgk et al. 2020), SuperFi‐Cas9 (Bravo et al. 2022), Sniper2L (Y. H. Kim et al. 2023), and Correct‐Cas9 (Sung et al. 2025), all designed to minimize off‐target activity. More recently, advances in artificial intelligence (AI) have further accelerated the engineering of CRISPR systems, exemplified by the development of de novo‐designed CRISPR nucleases using generative models (Ruffolo et al. 2025). Nevertheless, because target recognition is ultimately mediated by gRNA–DNA interactions, engineering of the guide RNA remains a complementary and highly versatile strategy for improving editing precision across diverse CRISPR platforms.

**FIGURE 1:**
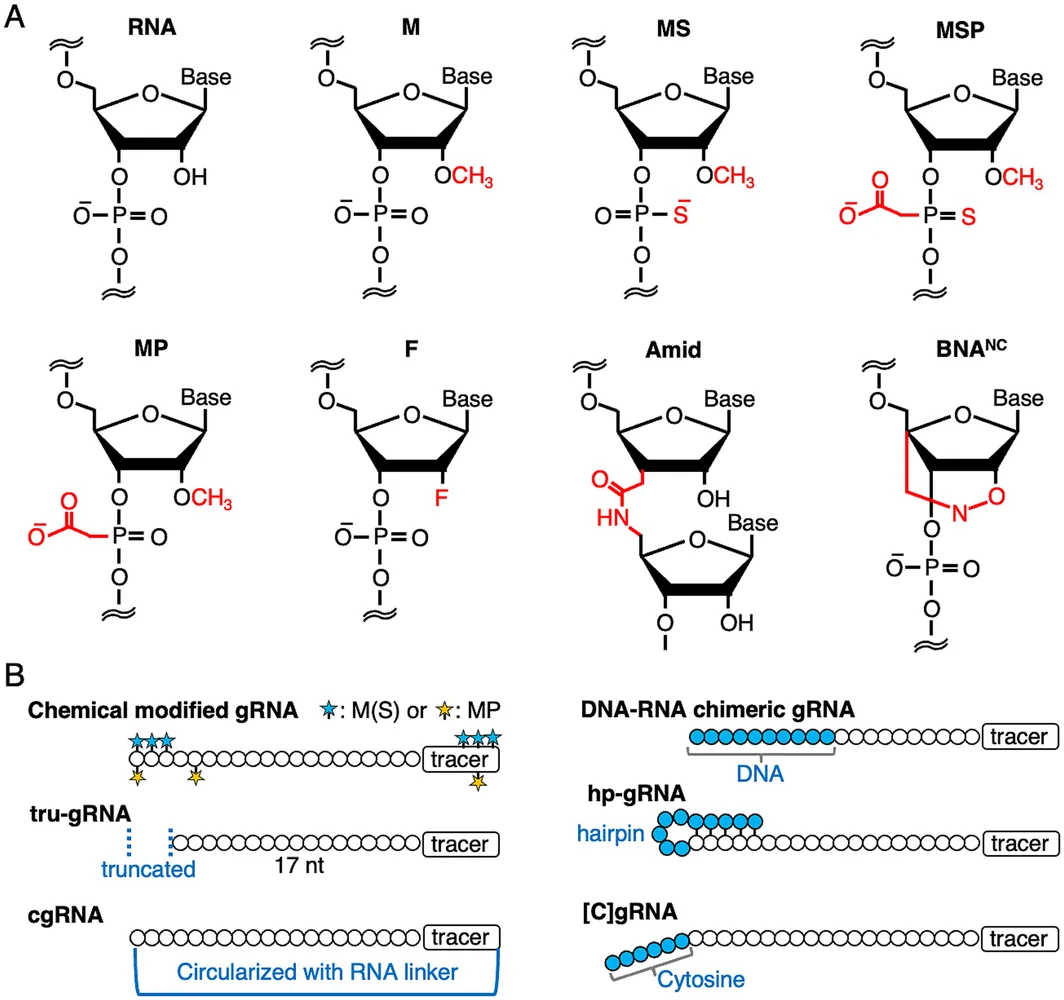
Overview of representative gRNA engineering strategies. (A) Representative chemical modifications introduced into gRNAs. M, 2′‐O‐methyl; MS, 2′‐O‐methyl phosphorothioate; MSP, 2′‐O‐methyl 3'thioPACE (2′‐O‐methyl 3′‐thiophosphonoacetate); MP, 2′‐O‐methyl 3′‐phosphonoacetate; F, 2′‐fluoro; Amid, amide backbone modification; BNA^NC^, N‐methyl‐substituted bridged nucleic acid analogue. These modifications primarily improve gRNA stability, specificity, nuclease resistance, and genome‐editing activity. (B) Schematic representation of major gRNA engineering approaches, including chemical modifications, spacer‐length optimization, and structural engineering. Representative examples include 2′‐O‐methyl (2′‐OMe)‐based modifications, DNA–RNA chimeric gRNAs, truncated gRNAs (tru‐gRNAs), hairpin gRNAs (hp‐gRNAs), circular gRNAs (cgRNAs), and cytosine‐extended‐gRNAs ([C]gRNAs). These strategies improve genome‐editing performance by enhancing gRNA stability, specificity, and programmable control of Cas activity.

In parallel, increasing attention has been directed towards strategies that optimise the gRNA itself to control editing outcomes. A variety of gRNA modifications have been explored, including chemical modification, partial replacement of RNA nucleotides with DNA, and alterations in spacer length or secondary structure (Figure 1A,B). Such modifications can enhance gRNA stability, reduce binding affinity to mismatched DNA, and thereby improve on‐target efficiency while reducing off‐target activity and cytotoxicity. Recent studies have developed gRNAs capable of fine‐tuning Cas9 activity (Kawamata et al. 2023). Importantly, combining distinct gRNA modifications holds the potential to achieve a balance of efficiency and safety that cannot be realised by single strategies alone. Moreover, the integrated use of high‐performance Cas9 variants together with rationally engineered gRNAs is expected to greatly accelerate the translation of CRISPR technology into clinical applications. Despite the rapid expansion of gRNA‐related studies, a comprehensive synthesis that unifies chemical, structural, and computational design strategies has been lacking. This review addresses that gap by integrating molecular design principles and translational perspectives relevant to genome‐editing therapeutics.

## 2 General Strategies for gRNA Design

At the outset of a genome‐editing experiment, it is essential to design a 20‐nucleotide spacer sequence that ensures high on‐target efficiency while minimising off‐target effects. Several web‐based design tools are currently used for this purpose (Table 1). These include Cas‐Designer (Park et al. 2015), CHOPCHOP v3 (Labun et al. 2019), CRISPick (MIT CRISPR design tool), and CRISPOR (Concordet and Haeussler 2018), which provide functionalities ranging from gRNA design and ranking to activity prediction and off‐target assessment.

**TABLE 1:** Representative computational tools for gRNA design and activity prediction.

Tool name	Primary function	Methodology	Web link
Cas‐Designer	gRNA design and off‐target prediction	Sequence alignment–based	rgenome.net/cas‐designer
CHOPCHOP v3	gRNA design and ranking	Rule‐based scoring and machine learning	chopchop.cbu.uib.no
CRISPOR	gRNA design and off‐target analysis	Integrated scoring algorithms	crispor.org
CRISPick	Optimized gRNA selection	Machine learning	portals.broadinstitute.org/gppx/crispick/public
CRISPRon	On‐target activity prediction	Deep learning	rth.dk/resources/crispr/crispron
DeepCRISPR	On‐target and off‐target prediction	Deep learning	github.com/bm2‐lab/DeepCRISPR
DeepHF	High‐fidelity gRNA activity prediction	Deep learning	deephf.com

*Note:* Representative computational tools used for guide RNA design, activity prediction, and off‐target assessment. Traditional sequence‐based approaches have been complemented by machine learning and deep learning models, enabling more accurate prediction of gRNA efficiency and specificity and facilitating the rational design of CRISPR genome‐editing experiments.

The computational prediction methods employed by these tools can be broadly classified into two categories (Kalter et al. 2025). The first is a sequence‐based approach, which scans the reference genome to identify candidate gRNAs that satisfy target‐site and PAM‐sequence requirements, while also detecting potential off‐target sites with sequence similarity to the intended target. In this case, sequence homology between the gRNA and genomic DNA is used to predict off‐target locations. The second is a scoring‐based approach, which includes hypothesis‐driven algorithms that incorporate known biological parameters, such as mismatch position or kinetic models, and machine learning (ML) algorithms trained and validated on experimental datasets to predict cleavage efficiency at both on‐target and off‐target sites. Recent advances in machine learning and deep learning (DL) have further improved the accuracy of gRNA activity prediction. Representative DL‐based models include CRISPRon (Anthon et al. 2022) for on‐target activity prediction, DeepCRISPR (Chuai et al. 2018) for integrated prediction of on‐target efficiency and off‐target propensity, and DeepHF (Wang et al. 2019) for activity prediction across both wild‐type and high‐fidelity Cas9 variants (Table 1). By automatically extracting complex sequence determinants from large experimental datasets, these models have significantly enhanced the accuracy of gRNA activity and specificity prediction compared with conventional rule‐based or scoring‐based methods.

Although ML‐based models grounded in experimental data appear highly reliable, their training datasets are often derived from specific cell types or experimental conditions and may not be directly generalisable to other contexts. In addition, unanticipated off‐target events frequently occur that escape predictive algorithms (Hoijer et al. 2020). In some cases, the intended target sequence resides in a genomic region that is intrinsically difficult to edit without off‐target activity. Therefore, it is crucial to experimentally validate gRNAs designed in silico and, if necessary, apply additional optimisation strategies such as chemical modifications or secondary structure alterations.

Building on these fundamental design principles, the following sections highlight several representative strategies of gRNA engineering that have proven effective in Cas9‐mediated genome editing.

## 3 Engineering of Synthetic gRNAs

Unlike vector‐expressed gRNAs, synthetic gRNAs can be engineered with a variety of chemical modifications at the single‐nucleotide level to enhance their performance. Representative examples of such modifications are introduced below.

### 3.1 Chemical Modifications of gRNAs

Synthetic gRNAs are rapidly degraded by cellular ribonucleases, making improved stability a major challenge. Introducing chemical modifications into the ribose sugar, phosphate backbone, or nucleobases of gRNAs increases nuclease resistance and prolongs their intracellular half‐life (Barber et al. 2025). A commonly used strategy involves applying 2′‐O‐methyl (2′‐OMe) modifications (M), which can be introduced at various positions throughout the gRNA to enhance stability and reduce immunogenicity. In particular, combining 2′‐OMe modifications with phosphorothioate (PS) linkages (MS) at one to three terminal nucleotides at both the 5′ and 3′ ends of the gRNA has become a widely adopted strategy for protecting gRNAs from nuclease‐mediated degradation (Hendel et al. 2015). Within this class of 2′‐OMe‐based modifications, 2′‐O‐methyl 3'thioPACE (MSP; 2′‐O‐methyl 3′‐thiophosphonoacetate) has also been developed to further enhance gRNA stability in primary cells (Hendel et al. 2015). MSP combines a 2′‐O‐methyl ribose modification with a thiophosphonoacetate backbone linkage, thereby increasing resistance to nuclease degradation while maintaining genome editing activity (Figure 1A, Table 2).

**TABLE 2:** Representative chemical engineering strategies of synthetic gRNAs and their functional effects.

Modification type	Category	Typical modification site	Functional effect
M (2′‐O‐Methyl (2′‐OMe))	Ribose modification	Termini/Internal positions	Increased stability and reduced immunogenicity
MS (2′‐OMe + PS)	Ribose/Backbone modification	Termini	Increased stability and activity
MSP (2′‐OMe + 3'thioPACE)	Ribose/Backbone modification	Termini	Increased stability and activity
MP (2′‐OMe + 3′‐phosphonoacetate)	Ribose/Backbone modification	Termini/Internal positions	Increased specificity with preserved activity
2′‐Fluoro (2′‐F)	Ribose modification	Spacer/tracrRNA	Increased stability and activity
Amide	Backbone modification	Backbone	Increased stability and specificity
5′ Capping (m^7^G)	5′ Terminal modification	5′ terminus	Increased stability and activity
LNA (locked nucleic acid)	Ribose modification	Spacer/tracrRNA	Increased stability and specificity
BNA^NC^ (bridged nucleic acid analogue)	Bridged nucleic acid analogue	Spacer/tracrRNA	Increased stability and specificity
DNA–RNA Chimeric gRNA	Nucleotide substitution	Spacer	Increased specificity

*Note:* Representative chemical modifications introduced into gRNAs to improve stability, specificity, nuclease resistance, target recognition, and genome‐editing efficiency.

Beyond improving gRNA stability, chemical modifications can also enhance genome‐editing specificity. Recent studies have demonstrated that backbone modifications at specific positions within the spacer region can markedly reduce off‐target cleavage while preserving strong on‐target activity. For example, introducing a single 2′‐O‐methyl‐3′‐phosphonoacetate (MP) modification within the spacer sequence substantially suppresses off‐target activity without compromising on‐target performance (Ryan et al. 2018). Thus, even a single backbone modification can tighten Cas9–DNA recognition and improve cleavage fidelity at the intended target site (Figure 1A, Table 2).

Additional chemical modalities have also been explored to further improve gRNA stability, nuclease resistance, and biocompatibility. For instance, 2′‐fluoro (2′‐F) substitutions at the ribose C2′ position increase thermal stability and resistance to intracellular RNases (Sakovina et al. 2022). Likewise, amide internucleoside linkages have been investigated as alternatives to the natural phosphodiester backbone to reduce susceptibility to enzymatic degradation (Kotikam et al. 2022) (Figure 1A, Table 2). Furthermore, 5′ capping strategies that append synthetic cap‐like structures containing 7‐methylguanosine (m^7^G) can protect gRNAs from cellular exonucleases and attenuate activation of innate immune sensors in sensitive cell types (Mu et al. 2019) (Table 2).

Another class of chemical modifications involves bridged nucleic acids (BNAs), including locked nucleic acids (LNAs) and bridged nucleic acid analogues (BNA^NCs^) (Cromwell et al. 2018) (Figure 1A, Table 2). BNAs contain a conformationally constrained ribose ring formed by a 2′‐O,4′‐C‐methylene bridge, which enhances duplex stability and nuclease resistance. BNA^NCs^ represent advanced derivatives of BNAs that incorporate an N‐methyl substitution within the bridging moiety. This modification increases conformational flexibility during nucleic acid binding while maintaining high nuclease resistance and reducing cytotoxicity (Rahman et al. 2008). Incorporation of BNA^NCs^ at multiple positions within the gRNA spacer sequence improves genome‐editing specificity by slowing Cas9 cleavage kinetics and reducing off‐target activity (Cromwell et al. 2018).

Collectively, these chemical modifications improve guide RNA performance through three principal mechanisms: increasing nuclease resistance, modulating Cas9–target interactions, and reducing immunogenicity.

### 3.2 DNA–RNA Chimeric gRNAs

Another approach to reducing off‐target activity is the use of DNA–RNA chimeric gRNAs, in which part of the spacer region is replaced by DNA nucleotides (Yin et al. 2018) (Figure 1B, Table 2). Substituting the spacer from the 5′ to the 3′ end with DNA can effectively suppress off‐target activity while maintaining on‐target editing efficiency when up to approximately 10 bases are replaced. This enhanced specificity is attributed to altered target recognition by the Cas9–gRNA complex following partial DNA substitution within the spacer region (H. Kim et al. 2020) (Yin et al. 2018). Replacement of RNA nucleotides with DNA reduces interactions between ribose 2′‐hydroxyl groups and the Cas9 protein, thereby decreasing stable binding to mismatched target sequences and enhancing cleavage fidelity (Yin et al. 2018). Longer DNA substitutions cause a length‐dependent loss of on‐target activity. Because the extent of this loss varies depending on the target sequence, the optimal DNA replacement region must be empirically determined for each target to maximise the on/off ratio.

## 4 Structural Engineering of gRNAs

In expression‐based genome‐editing approaches that rely on cellular transcription, such as those employing adeno‐associated virus (AAV), both Cas proteins and gRNAs are expressed endogenously within cells. Under these conditions, the direct incorporation of chemical modifications or DNA nucleotides into gRNAs is generally not feasible. Consequently, optimization strategies have focused on engineering gRNA length, sequence composition, and secondary structure to enhance editing performance. These structural design principles are critical not only for intracellularly expressed gRNAs but also for optimizing synthetic gRNAs (Nowak et al. 2016). Representative examples of such structural engineering approaches are discussed below.

### 4.1 Truncated gRNAs (Tru‐gRNAs)

Truncated gRNAs (tru‐gRNAs) consist of shortened spacer regions, typically 17–18 nucleotides rather than the conventional 20 (Fu et al. 2014) (Figure 1B, Table 3). This shortening increases Cas9's sensitivity to mismatches, leading to a marked reduction in off‐target editing. When the spacer length is shortened to approximately 17 nucleotides, on‐target activity is largely preserved in many contexts, making tru‐gRNAs a simple yet effective strategy to improve specificity. However, depending on the target sequence, substantial reductions in activity can occur, highlighting the need to optimise spacer length individually for each target to maximise the on/off‐target ratio.

**TABLE 3:** Representative structural engineering strategies of gRNA and their functional effects.

Modification type	Structural feature	Functional effect
Truncated gRNA (tru‐gRNA)	Shortened spacer (17–18 nt)	Increased specificity
Hairpin gRNA (hp‐gRNA)	5′ terminal hairpin structure	Increased specificity
[C]gRNA	5′ cytosine extension	Tunable Cas9 activity with increased specificity and safety
Circular gRNA (cgRNA)	Covalently closed circular structure	Increased stability and activity
Topology‐engineered gRNA	Photoresponsive, cross‐linked, or multivalent topology	Programmable spatiotemporal control of CRISPR activity
tracrRNA Engineering	Sequence and secondary‐structure engineering	Tunable Cas9 binding, activity, and stability
G‐quadruplex (G4) gRNA	G‐quadruplex‐forming motif at 3′ end of tracrRNA	Increased stability and editing efficiency

*Note:* Representative structural engineering strategies that improve genome‐editing performance by modulating spacer length, RNA topology, scaffold architecture, and secondary structure to enhance specificity, stability, activity, safety, and spatiotemporal control.

Further shortening of the spacer region to ~15 nucleotides abolishes on‐target activity almost completely. Interestingly, this “inactivation” property has been exploited in the development of the CRISPR GUARD strategy, in which tru‐gRNAs are directed to specific off‐target loci to competitively block binding of the Cas9:(20 nt)gRNA complex and thereby suppress unwanted editing (Coelho et al. 2020). Although this method requires the use of multiple gRNAs and can only suppress a limited subset of off‐target sites, CRISPR GUARD represents a promising approach for mitigating off‐target effects.

### 4.2 Hairpin gRNAs (hp‐gRNAs)

Hairpin gRNAs (hp‐gRNAs) were developed to improve editing specificity by modifying the secondary structure of the spacer region (Kocak et al. 2019) (Figure 1B, Table 3). This approach involves appending a short sequence at the 5′ end that is complementary to the spacer, thereby forming a hairpin structure upstream of the spacer. The resulting steric hindrance interferes with R‐loop formation between the gRNA and target DNA, leading to a significant reduction in cleavage activity at off‐target sites. Notably, hp‐gRNAs can substantially suppress off‐target activity while maintaining on‐target performance. In some cases, however, on‐target efficiency is reduced depending on the sequence context. This limitation can be mitigated by adjusting the length of the complementary sequence to optimise the on/off‐target ratio.

### 4.3 Circular and Topology‐Engineered gRNAs

Another strategy for engineering gRNA secondary structure involves the development of circular gRNAs (cgRNAs) (Liu et al. 2023; Zhang et al. 2025, 2023) (Figure 1B, Table 3). cgRNAs are generated by covalently linking the two ends of a linear single‐stranded gRNA with an insert sequence, thereby forming a closed circular structure. Compared with linear RNAs, cgRNAs exhibit enhanced nuclease resistance and reduced susceptibility to intracellular degradation. Consequently, they remain functional for extended periods and can enhance the editing efficiency of Cas9 and base editors.

Two main approaches for cgRNA synthesis have been described: ligation of in vitro–synthesized linear gRNAs (Liu et al. 2023) and intracellular circularisation of plasmid‐expressed gRNAs via self‐splicing ribozymes and endogenous RNA ligases (Zhang et al. 2025; Zhang et al. 2023). The latter strategy is particularly applicable to AAV‐based therapeutic systems.

Recent studies have further expanded this concept beyond simple circularization toward broader topology engineering of gRNAs. Representative examples include photoresponsive cyclic gRNAs (Sun et al. 2023), conditionally activated cross‐linked crRNAs (Chen et al. 2025a), and star‐shaped multivalent crRNAs (Chen et al. 2025b), all of which regulate CRISPR activity by manipulating higher‐order RNA topology rather than spacer sequence alone. Unlike conventional nucleotide modifications that primarily improve stability or nuclease resistance, these topology‐engineering strategies enable programmable regulation of CRISPR activity. Together, these advances establish topology engineering as a versatile strategy for achieving precise spatiotemporal and functional control of CRISPR systems.

Despite these advances, rational design principles for topology‐engineered gRNAs remain incompletely understood. With respect to circular gRNAs, a broad range of insert lengths and sequence architectures has been reported to be compatible with circular gRNA construction, although the optimal length and sequence composition remain to be determined. Future progress is expected through AI‐driven design frameworks capable of optimizing insert sequences according to individual Cas proteins, guide RNA architectures, and spacer contexts, thereby facilitating the rational design of next‐generation topology‐engineered gRNAs.

### 4.4 Cytosine‐Extended gRNAs ([C]gRNAs)

Cytosine‐extended gRNAs ([C]gRNAs), in which cytosine residues are added to the 5′ end of the spacer, represent a simple yet effective strategy for fine‐tuning Cas9 activity (Kawamata et al. 2023) (Figure 1B, Table 3). This approach is particularly valuable in situations where excessive Cas9 activity leads to undesirable outcomes such as off‐target editing or DSB‐induced cytotoxicity, thereby functioning as a safeguard gRNA. A key feature of [C]gRNAs is their length‐dependent ability to suppress Cas9 activity through three primary mechanisms: reduced binding affinity of the gRNA–Cas9 complex to target DNA, impaired DNA‐cleavage capacity, and decreased transcriptional efficiency of the gRNA under the U6 promoter. Each of these mechanisms operates in a [C]‐length‐dependent manner.

Short [C]gRNAs (e.g., 5C) retain sufficient on‐target activity for biallelic knockout while only slightly reducing cleavage efficiency. At the same time, they markedly lower off‐target events and p53‐dependent cytotoxicity and can further enhance the efficiency of HDR. In contrast, longer [C] extensions (e.g., 20C) reduce on‐target activity but increase allele‐specific editing, enabling precise monoallelic repair in which only the disease‐associated SNP‐containing allele is corrected by HDR while the wild‐type allele is preserved.

Moreover, simulation models have been developed to predict the relationship between Cas9 activity, editing efficiency, and safety. These models support the rational selection of optimal [C]gRNAs and offer the potential to implement advanced editing strategies directly applicable to clinical settings.

### 4.5 Engineering tracrRNA

The tracrRNA is a critical component that anchors the gRNA to Cas9 through its structured stem‐loops and plays an essential role in the assembly and activity of the Cas9–gRNA complex (Jinek et al. 2012; Nishimasu et al. 2014). Consequently, engineering of the tracrRNA has emerged as an effective strategy to improve gRNA stability, genome‐editing efficiency, and regulatory functionality. Although numerous tracrRNA engineering approaches have been reported, only representative examples are highlighted here.

Recent studies have demonstrated that rational engineering of the tracrRNA scaffold can improve Cas9–gRNA assembly, stability, and genome‐editing activity through optimization of guide RNA architecture (Barber et al. 2025). For example, extension of the tracrRNA–crRNA duplex together with disruption of the RNA polymerase III termination signal within the sgRNA scaffold enhances Cas9 binding and genome‐editing efficiency (Dang et al. 2015), illustrating how scaffold engineering can improve ribonucleoprotein assembly without altering spacer‐mediated target recognition.

Notably, systematic engineering of tracrRNA sequences has been shown to generate guide RNAs with graded activities, enabling precise tuning of gene regulation without altering target‐site recognition (Jost et al. 2020) (Table 3).

Another example involves incorporation of G‐quadruplex (G4)‐forming motifs into the tracrRNA region. G4 structures are compact secondary structures formed by guanine‐rich sequences and are thought to protect gRNAs from exonuclease‐mediated degradation. Introduction of G4 motifs enhances intracellular gRNA stability and editing efficiency without altering spacer‐mediated target recognition (Nahar et al. 2018) (Table 3).

Together, these studies demonstrate that rational engineering of the tracrRNA can modulate gRNA activity, stability, and functional performance while preserving target specificity.

## 5 Clinical and Translational Applications of Chemically Modified gRNAs

Although numerous gRNA engineering strategies have been developed, relatively few studies have disclosed the exact chemical compositions of gRNAs used in therapeutic applications. Representative examples are highlighted below.

A prominent example is exagamglogene autotemcel (exa‐cel; Casgevy), the first approved CRISPR–Cas9 therapy for sickle cell disease and transfusion‐dependent β‐thalassaemia (Frangoul et al. 2021). In this approach, autologous CD34+ hematopoietic stem and progenitor cells are edited ex vivo at the erythroid enhancer of *BCL11A* using Cas9 ribonucleoproteins (RNPs). The 100‐mer gRNA contains three terminal MS‐modified nucleotides at both the 5′ and 3′ termini, corresponding to the widely adopted 2′‐OMe/PS stabilization strategy described in Section 3.1.

Another important example is renizgamglogene autogedtemcel (reni‐cel), an investigational CRISPR–Cas12a therapy for sickle cell disease and transfusion‐dependent β‐thalassaemia. In this ex vivo gene‐editing approach, autologous CD34+ hematopoietic stem and progenitor cells are edited at the distal CCAAT box region of the *HBG1* and *HBG2* promoters to disrupt BCL11A‐mediated repression and induce fetal hemoglobin expression. Although the precise chemical composition of the gRNA has not been disclosed, the gRNA used in this study was chemically synthesized and designed to target the distal CCAAT box region of the *HBG1* and *HBG2* promoters. This example extends the clinical application of gRNA technologies beyond Cas9 to the CRISPR–Cas12a platform.

A second example is CTX310, an in vivo CRISPR–Cas9 therapy targeting *ANGPTL3* for the treatment of dyslipidaemia (Laffin et al. 2025). In this system, Cas9 mRNA and a chemically modified gRNA are delivered to hepatocytes using lipid nanoparticles (LNPs). The 100‐mer gRNA contains 48 2′‐OMe‐modified nucleotides together with three terminal PS linkages at both the 5′ and 3′ termini, representing a substantially higher degree of chemical modification than that reported for Casgevy.

Another notable example is the recently reported personalized base‐editing therapy for carbamoyl phosphate synthetase 1 (*CPS1*) deficiency in a newborn infant (Musunuru et al. 2025). This study represents a landmark example of personalized in vivo genome‐editing therapy tailored to an individual patient. In this study, a patient‐specific adenine base editor was delivered to the liver using LNPs to correct a pathogenic *CPS1* mutation. The guide RNA drug substance (kayjayguran) was a 100‐mer oligonucleotide containing 47 2′‐OMe ribonucleotides and three terminal PS linkages at both the 5′ and 3′ ends.

Collectively, these examples demonstrate that chemically engineered or chemically synthesized gRNAs have already become integral components of therapeutic genome‐editing platforms. Although the extent and precise composition of chemical modification varies among applications, currently disclosed clinical and translational programs consistently employ chemically optimized gRNAs to improve stability, activity, and in vivo performance. These examples further illustrate that the optimal extent and type of gRNA engineering should be selected according to the delivery platform, CRISPR system, and therapeutic modality rather than following a universal design strategy.

Beyond therapeutic genome editing, CRISPR‐based molecular diagnostic platforms such as SHERLOCK and DETECTR have emerged as powerful tools for highly sensitive nucleic acid detection (Chen et al. 2018; Gootenberg et al. 2017). More recently, chemical and structural engineering of guide RNAs has been explored to further improve these platforms by enhancing nuclease resistance, minimizing nonspecific activation, and enabling programmable temporal control of CRISPR activity (Zou et al. 2026). Such advances have expanded the applicability of CRISPR diagnostics to clinically relevant settings, including the detection of infectious diseases such as SARS‐CoV‐2, HBV genotyping, and cancer‐derived circulating tumor DNA (ctDNA), highlighting the growing translational potential of guide RNA engineering in precision diagnostics (Zou et al. 2026).

## 6 Conclusion and Future Perspectives

gRNA engineering has evolved from a simple optimization strategy into a central determinant of CRISPR performance, influencing editing efficiency, specificity, stability, safety, and controllability. As summarized in this review, diverse chemical, structural, and computational approaches provide complementary strategies for improving genome‐editing outcomes across a broad range of CRISPR applications. Rather than serving merely as auxiliary components of the CRISPR system, gRNAs are increasingly recognized as programmable engineering targets that can be rationally optimized according to specific therapeutic and diagnostic requirements.

Importantly, the optimal strategy for gRNA engineering depends on the mode of guide RNA delivery. In applications utilizing synthetic gRNAs, including ex vivo editing and lipid nanoparticle LNP‐mediated in vivo delivery, chemical modifications such as 2′‐O‐methyl and phosphorothioate linkages have already become integral components of clinically translated genome‐editing therapies, as exemplified by Casgevy, reni‐cel, CTX310, and personalized base‐editing programs. These modifications improve nuclease resistance, reduce immunogenicity, and enhance genome‐editing efficiency, thereby facilitating the clinical translation of CRISPR therapeutics. In contrast, viral vector–based platforms, particularly AAV‐mediated systems, rely predominantly on intracellular transcription of gRNAs, making direct incorporation of chemical modifications impractical. Under these conditions, structural engineering strategies—including spacer optimization, topology engineering, scaffold engineering, and conformational regulation—are expected to play increasingly important roles in improving editing precision, activity, and biosafety. Accordingly, chemical and structural engineering should be regarded as complementary rather than competing approaches, with their selection guided by the delivery platform and therapeutic modality.

Beyond therapeutic genome editing, gRNA engineering is also emerging as a key enabling technology for CRISPR‐based molecular diagnostics and biosensing. Rational engineering of gRNAs has been shown to improve nuclease resistance, assay robustness, and programmable control of CRISPR activity, thereby expanding the clinical applicability of platforms such as SHERLOCK and DETECTR. Together with advances in therapeutic genome editing, these developments underscore the broad translational potential of gRNA engineering across precision medicine.

Looking forward, the integration of gRNA engineering with AI‐assisted design, high‐fidelity CRISPR nucleases, advanced delivery technologies, and large‐scale functional datasets will enable increasingly precise and context‐dependent optimization of genome‐editing systems. Future efforts should focus not only on improving individual engineering strategies but also on establishing unified design principles that integrate chemical, structural, and computational approaches according to specific clinical applications. Ultimately, the co‐evolution of engineered Cas proteins and engineered gRNAs will provide a foundation for developing safer, more precise, and clinically translatable genome‐ and epigenome‐editing technologies.

## Author Contributions

**Atsushi Suzuki:** writing – review and editing, supervision, writing – original draft. **Satoshi Niwa:** writing – review and editing. **Masaki Kawamata:** conceptualization, supervision, funding acquisition, project administration, writing – original draft, writing – review and editing.

## Funding

This work was supported in part by JSPS KAKENHI grants (JP18H04737, JP20H05041, JP23K18097, JP23K27462, and JP25K22439 to M.K.; JP18H05102, JP19H01177, JP19H05267, JP20H05040, JP22H05634, JP22H04698, JP22H00592, JP23K18579, JP25K22908, and JP25H00445 to A.S.), the AMED (JP18ae0201012, JP24ek0109708, and JP25nk0101727 to M.K.; JP24fk0210116, JP24bm1123005, and JP25fk0210170 to A.S.), the MEXT Promotion of Development of a Joint Usage/Research System Project: Coalition of Universities for Research Excellence Program (JPMXP1323015486 to A.S.) and Cooperative Research Project Program (to A.S.), the Center for Clinical and Translational Research of Kyushu University Hospital (to M.K.), the Fukuoka Financial Group Enterprise Development Foundation (KYUTEC) (to M.K.), the Medical Research Center Initiative for High Depth Omics (to M.K. and A.S.), the Takeda Science Foundation (to M.K., and A.S.), the Uehara Memorial Foundation (to A.S.), and the Naito Foundation (to A.S.).

## Conflicts of Interest

M.K. is a co‐founder and Chief Scientific Officer of One Genomics Inc. and holds equity in the company. The remaining authors declare no conflicts of interest.

## Data Availability

The authors have nothing to report.
